# Mouse Model for Assessing the Subchronic Toxicity of Organophosphate Pesticides

**Published:** 2018

**Authors:** V. A. Palikov, S. S. Terekhov, Yu. A. Palikova, O. N. Khokhlova, V. A. Kazakov, I. A. Dyachenko, S. V. Panteleev, Yu. A. Mokrushina, V. D. Knorre, O. G. Shamborant, I. V. Smirnov, A. G. Gabibov

**Affiliations:** Branch of the Institute of Bioorganic Chemistry, Academicians M.M. Shemyakin and Yu.A. Ovchinnikova RAS, Nauki Ave., 6, Pushchino, Moscow region, 142290, Russia; Institute of Bioorganic Chemistry, Academicians M.M. Shemyakin and Yu.A. Ovchinnikova RAS, Miklukho-Maklaya Str., 16/10, Moscow, 117997, Russia; Faculty of Chemistry, Moscow State University M.V. Lomonosov, Leninskie gori, 1, bldg. 3, Moscow, 119991, Russia

**Keywords:** butyrylcholinesterase, in vivo model, organophosphorus toxins, bioscavenger

## Abstract

The development of antidotes to organophosphate poisons is an important aspect of modern pharmacology. Recombinant acetylcholinesterase and butyrylcholinesterase are effective DNA-encoded acceptors of organophosphate poisons and, in particular, pesticides. Here, we present the results of a study on the effectiveness of recombinant butyrylcholinesterase (BChE) in modeling organophosphate poisoning caused by oral administration of paraoxon at a dose of 2 mg / kg. The study showed a high activity of BChE as a protective agent for subchronic anticholinesterase poisoning in an *in vivo *model. The administration of BChE in a dose of 20 mg / kg allows one to avoid mortality, and also contributed to rapid recovery after model poisoning.

## INTRODUCTION

The modern therapy of acute and severe chronic poisoning with organophosphorus agents (OPs) involves resuscitation, mechanical ventilation, treatment with a muscarinic antagonist (typically, atropine), in combination with the administration of large amounts of liquid and an acetylcholinesterase activator (e.g., pralidoxime) [1]. However, such treatment often causes severe adverse events: nausea, vomiting, and partial or total disability as it is impossible to avoid the risk of irreversible neuronal damage.

Application of biological antidotes, biomolecules that bind to OPs and inactivate them, is one of the promising approaches to the treatment of organophosphate poisoning [2-5]. Such an enzyme as human butyrylcholinesterase (hBChE) and antibodies capable of binding to OPs or hydrolyzing them are regarded today as potential bioscavengers [6, 7]. hBChE is a natural biological antidote (a suicidal inactivator) for organophosphate poisoning. Due to its unique similarity to human acetylcholinesterase (hAChE) and the large volume of the cavity in its active site, hBChE inactivates a broad range of OPs and is often even more efficient than hAChE [8]. Furthermore, application of hBChE allows one to avoid the long-term adverse effects of OP poisoning, including irreversible brain damage [9].

Organophosphorus agents form the largest group of chemical pesticides used for plant protection. Since most people eat fresh fruits and vegetables, they automatically belong to the group of people susceptible to an increased risk of pesticide poisoning. Paraoxon, an active metabolite of the parathion pesticide, is considered one of the most potent agents that can inhibit hAChE [10]. Paraoxon and insecticides similar to paraoxon penetrate an organism through skin contact or the gastrointestinal tract [11], which leads to acute or chronic poisoning in humans and animals. Furthermore, most OP-based insecticides are lipophilic agents that are prone to accumulation in adipose tissues, which significantly increases the potential for a chronic effect on the human organism. Hence, the development of *in vivo *models making it possible to evaluate the subchronic toxicity of organophosphorus pesticides is of substantial interest, since it allows one to identify the long-term effects of exposure to OPs on animal’s physiological and behavioral characteristics.

## MATERIALS AND METHODS

Toxicity of rhBChE was studied in 36 BALB/c mice. The mice were allocated into three groups (two study groups and one control group), with six males and six females per group. Formation of these groups allows one to obtain a representative sample and statistically significant data. Prior to study initiation, the groups of animals in cages were placed in a separate room and left there for 7 days for adaptation. The signs of abnormalities in animals’ health were monitored during this period. Healthy animals with an individual weight corresponding to the mean weight for the respective sex with 10% accuracy were randomly selected to be used in the experiment. The main guidelines for animal housing and care complied with the regulations listed in the Guide for Care and Use of Laboratory Animals (ILAR publication, 1996, National Academy Press).

The animals in the study group received a subcutaneous injection of a carboxylesterase inhibitor, cresylbenzodioxaphosphorin oxide (CBDP), at a dose of 1.5 mg/kg. Fifteen minutes later, the mice were given an intravenous injection of either rhBChE at a dose of 20 mg/kg or normal saline and subsequently received an oral dose of paraoxon (2 mg/kg). The agents were administered on study days 1, 3, and 5. An integrated testing was conducted after the third administration, on study day 6. An animal’s body weight, food, and water intake were measured daily. Performance tests, such as grip strength test, assessment of respiratory parameters, and locomotor and exploratory activity in the animals were carried out to evaluate antidote effectiveness.

**Recording respiratory parameters**

The status of the respiratory system was assessed using the PowerLab 8/35 software. Such parameters as the respiratory rate (breaths per minute), tidal volume (mL), and the peak expiratory flow (mL/s) were evaluated through this test. The test was performed on study day 6 (after the third administration of agents).

**Recording locomotor and exploratory activity**

Total locomotor and exploratory activity was recorded during integrated testing of the animals after clinical examination. Behavioral activity was analyzed using the open-field test on a TSE Multi-Conditioning System Extended Advanced multiple-purpose platform. The test was performed on study day 6 (after the third administration of the agents). Test duration was 3 min. An animal was placed into the “open field” of an actometer, and such parameters as the distance travelled (cm), immobility time (s), and the number of rearings was recorded.

**Recording muscle strength, which represents the function of peripheral nerves in the grip strength test**

The animal’s muscle strength was measured using a grip strength meter (Columbus Instruments). The force applied to the dynamometer grid by the animal’s front paws (kg) was recorded. The measurements were carried out during the integrated testing of the animal, after the procedure of recording locomotor activity, on study day 6 (after the third administration of the agent). Descriptive statistics were used for all the quantitative data obtained throughout the study. The Kruskal–Wallis one-way analysis of variance and/or the Mann–Whitney test were used to determine intergroup differences and to compare the study groups to the control one. Statistical analysis was carried out using the Statistica for Windows 7.1 software. The differences were regarded as statistically significant at *P* < 0.05. The results were presented as the value ± standard deviation (*P *≤ 0.0005).

## RESULTS AND DISCUSSION

**Fig. 1:**
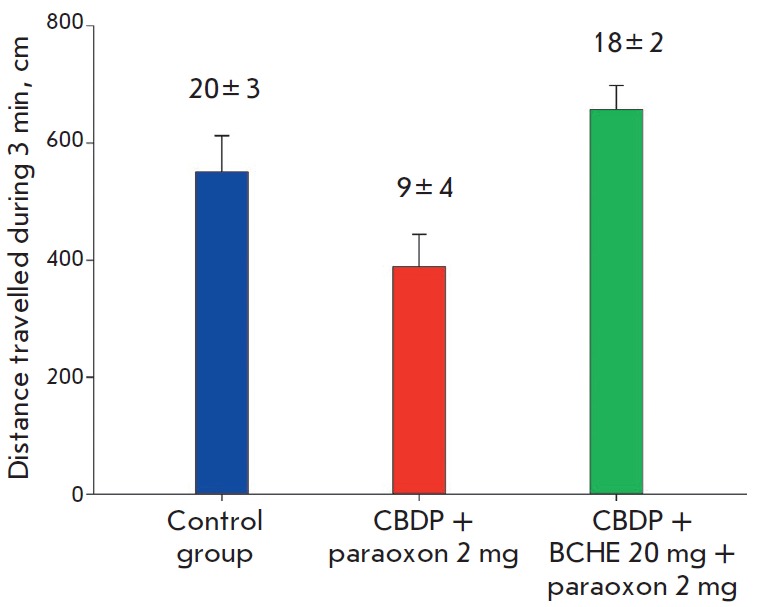
Analysis of locomotor and exploratory activity. The distance travelled by the animal (cm) was evaluated. The numbers above the respective bars correspond to the number of rearings. The error bars illustrate the standard deviation in a group

A biological model taking into account the difference between the “esterase” statuses of humans and mice has been elaborated to evaluate the effectiveness of butyrylcholinesterase as a therapeutic agent used to prevent organophosphate poisoning. The blood level of BChE in humans is twice as high as that in mice (5 and 2.6 mg/L, respectively), while the blood level of hAChE is 25-fold lower (0.008 and 0.2 mg/L, respectively). Furthermore, the conventionally used laboratory animals (rodents: mice, rats, and guinea pigs) have another evolutionarily important protection mechanism against OP poisoning. This mechanism is related to the presence of the carboxylesterase ES1 gene encoding an enzyme that irreversibly inactivates a broad range of OPs. Human blood plasma does not contain this enzyme, so the data can be misinterpreted when assessing the toxicity of OPs. There are two main esterases in human blood plasma: butyrylcholinesterase (hBChE, 5 mg/L) and PON1 (50 mg/L). In order to maximally reduce the background activity of endogenous carboxylesterase in mouse blood plasma, we used a specific inhibitor, cresylbenzodioxaphosphorin oxide (CBDP), at a dose of 1.5 mg/kg, which fully inhibited the activity of this enzyme. CBDP had been administered subcutaneously before the animal received an organophosphorus agent. Paraoxon was chosen as a model OP, since this agent and its analogues are natural metabolites of the overwhelming majority of the currently used organophosphorus pesticides. Chronic poisoning was simulated by oral administration of paraoxon, mimicking pesticide penetration into the organism during food consumption.

**Fig. 2:**
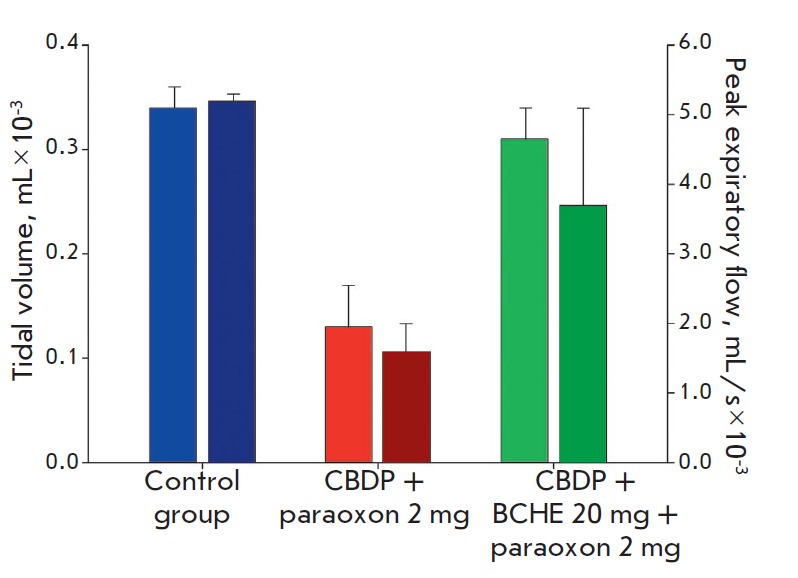
Analysis of respiratory parameters. The tidal volume (left columns) and the peak expiratory flow (right columns) were estimated. Testing was performed on study day 6 (after the 3^rd^ injection of agents). The error bars illustrate the standard deviation in a group

**Fig. 3:**
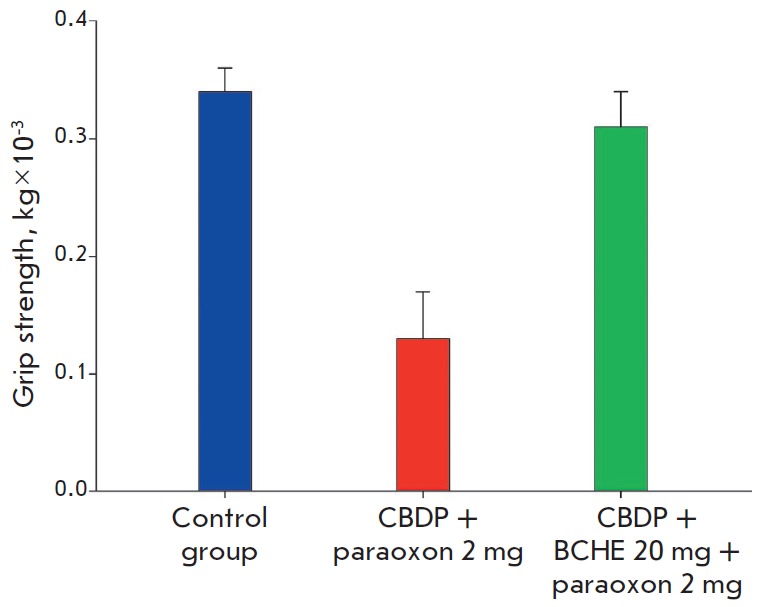
Analysis of muscle strength in the grip strength test. The force applied to the dynamometer grid from the animal’s front paws was recorded (kg). Error bars illustrate the standard deviation in a group

We demonstrated that locomotor activity fell in mice that received the OP without therapy with rhBChE. The distance travelled by the mice changed 1.5-fold; their exploratory activity decreased more than twofold (*Fig. 1*). In turn, administration of rhBChE completely restored the motor function and exploratory activity. The significant reduction in motor function and exploratory activity in our model was associated with strong suppression of respiratory center activity (*Fig. 2*). The key characteristics of respiratory function, such as tidal volume and the peak expiratory flow, in the group of animals that received OP dropped threefold compared to those in the control group. Meanwhile, treatment with rhBChE helped recover normal respiration. A comparable effect was observed for grip strength (*Fig. 3*). Paraoxon significantly reduced muscle strength. Grip strength in animals that received the OP was 2.5-fold lower than that in the control group. Identically to the effects described earlier, treatment with rhBChE made it possible to maintain muscle activity and prevented the physiological manifestations of chronic exposure to paraoxon.

## CONCLUSIONS

Hence, we have elaborated a biological model that allows one to evaluate the subchronic toxicity of a paraoxon pesticide administered orally. This model is of significant interest in studying the chronic effects of exposure to OPs. It was demonstrated that key physiological characteristics, such as locomotor and exploratory activity, respiration, and muscle activity, were the parameters sensitive to OPs in our *in vivo *model. The rhBChE used as a protective agent exhibited high activity. Intravenous administration of this biological product at a dose of 20 mg/kg both prevented animal mortality and contributed to rapid recovery from poisoning. We found that the key physiological characteristics of animals treated with rhBChE did not differ from those in the control group not exposed to the toxic activity of paraoxon. This demonstrates that the biological product has a high protective effect not only for the earlier described acute toxicity model, but also for the developed biological model of subchronic toxicity.

The reduction in motor function, exploratory activity, respiratory parameters, and muscle strength reported for this biological model may be indicative of a loss of neuronal associations. A detailed evaluation of neurophysiological characteristics and the irreversibility of the effect of OPs for the elaborated biological model of subchronic toxicity is of significant interest and will be performed in further studies.
